# Trends in bednet ownership and usage, and the effect of bednets on malaria hospitalization in the Kilifi Health and Demographic Surveillance System (KHDSS): 2008–2015

**DOI:** 10.1186/s12879-017-2822-x

**Published:** 2017-11-15

**Authors:** Alice Kamau, Victoria Nyaga, Evasius Bauni, Benjamin Tsofa, Abdisalan M. Noor, Philip Bejon, J. Anthony G. Scott, Laura L. Hammitt

**Affiliations:** 1 KEMRI-Wellcome Trust Research Programme, CGMR-Coast, Kilifi, Kenya; 2 KEMRI-Wellcome Trust Research Programme, Nairobi, Kenya; 3 Department of Infectious Disease Epidemiology, London School of Hygiene & Tropical Medicine, London, UK; 4 Centre for Tropical Medicine and Global Health, Nuffield Department of Clinical Medicine, University of Oxford, CCVTM, Oxford, UK; 5 Department of International Health, Johns Hopkins Bloomberg School of Public Health, Baltimore, MD USA

**Keywords:** Malaria, Bednet, Kenya, Demographic Surveillance System

## Abstract

**Background:**

Use of bednets reduces malaria morbidity and mortality. In Kilifi, Kenya, there was a mass distribution of free nets to children < 5 years in 2006. In 2009, a new policy was implemented to offer bednets to pregnant women and children < 5 years free of charge. Nets were again distributed to children and adults through national mass campaigns in 2012 and 2015. We aimed to evaluate trends in bednet ownership and usage, and the effect of bednets on the incidence of malaria hospitalization in children < 5 years within the Kilifi Health and Demographic Surveillance System (KHDSS).

**Methods:**

Bednet ownership and usage were assessed during eight routine enumeration rounds of the KHDSS between 2008 and 2015. Malaria admissions (i.e. admissions to hospital with *P. falciparum* > 2500 parasitemia per μl) among children < 5 years were captured using a system of continuous vital registration that links admissions at Kilifi County Hospital to the KHDSS population register. Survival analysis was used to assess relative risk of hospitalization with malaria among children that reported using a bednet compared to those who did not.

**Results:**

We observed 63% and 62% mean bednet ownership and usage, respectively, over the eight-survey period. Among children < 5 years, reported bednet ownership in October–December 2008 was 69% and in March–August 2009 was 73% (*p* < 0.001). An increase was also observed following the mass distribution campaigns in 2012 (62% in May–July 2012 vs 90% in May–October 2013, *p* < 0.001) and 2015 (68% in June–September 2015 vs 93% in October–November 2015, *p* < 0.001). Among children <5 years who reported using a net the night prior to the survey, the incidence of malaria hospitalization per 1000 child-years was 2.91 compared to 4.37 among those who did not (HR = 0.67, 95% CI: 0.52, 0.85 [*p* = 0.001]).

**Conclusion:**

On longitudinal surveillance, increasing bednet ownership and usage corresponded to mass distribution campaigns; however, this method of delivering bednets did not result in sustained improvements in coverage. Among children < 5 years old bednet use was associated with a 33% decreased incidence of malaria hospitalization.

**Electronic supplementary material:**

The online version of this article (10.1186/s12879-017-2822-x) contains supplementary material, which is available to authorized users.

## Background

Malaria remains a global health problem with an annual incidence of 214 million cases and 438,000 deaths; more than 88% of the total morbidity is in the WHO Africa region [1–4]. Bednets have been demonstrated to reduce malaria-related morbidity and mortality in sub-Saharan Africa [3, 5–8]. Models suggest that, the proportion of the population who had access to bednets increased from 4 to 67% between 2004 and 2015 in sub-Saharan Africa [9]. WHO has recommended universal coverage of bednets (defined as 1 bednet per two persons) because of the effectiveness of bednets in malaria prevention [10–12]. Centralized mass distribution campaigns have been the cornerstone of the effort to achieve universal bednet coverage [10].

In Kilifi, Kenya, bednet coverage was estimated to be less than 6% in the early 1990s [13]. A randomized controlled trial of bednets was conducted in part of Kilifi in 1993, where bednets were distributed to residents in a defined geographical area representing approximately 30% of the population. Bednet use in other areas remained low. The trial found that bednet use was associated with a 33% reduction in childhood mortality and a 44% reduction in severe malaria morbidity [14]. In 2005, subsidized nets were made available to children and pregnant women through maternal and child health clinics, and in September 2006, a free bednet distribution campaign by the then Kilifi District Health Management Team increased coverage across Kilifi county from about 0.25 to 0.5 insecticide-treated nets per person [15]. After the campaigns, bednets continued to be provided to the community through maternal and child health clinics at a cost of 50 Kenyan Shillings (~ $0.50). This was the case until January 2009, when a new policy was implemented to offer bednets at government health clinics to pregnant women and children aged < 5 years free of charge and to the rest of the population for 50 Kenyan Shillings. Bednets were again distributed through mass campaigns in July 2012 and in October 2015 as part of the efforts of achieving universal coverage for people at risk of malaria.

Household possession of bednets is an indicator of the extent to which distribution channels are enhancing coverage. However, use of bednets, rather than possession, is what affords protection and is therefore a more valid predictor of epidemiological impact [16, 17]. The objective of this analysis was to evaluate trends in ownership and usage of bednets in the context of mass distribution campaigns, and to assess the effect of bednet ownership and usage on the incidence of hospitalized malaria under routine, non-trial conditions.

## Methods

### Study area and population

**Fig. 1:**
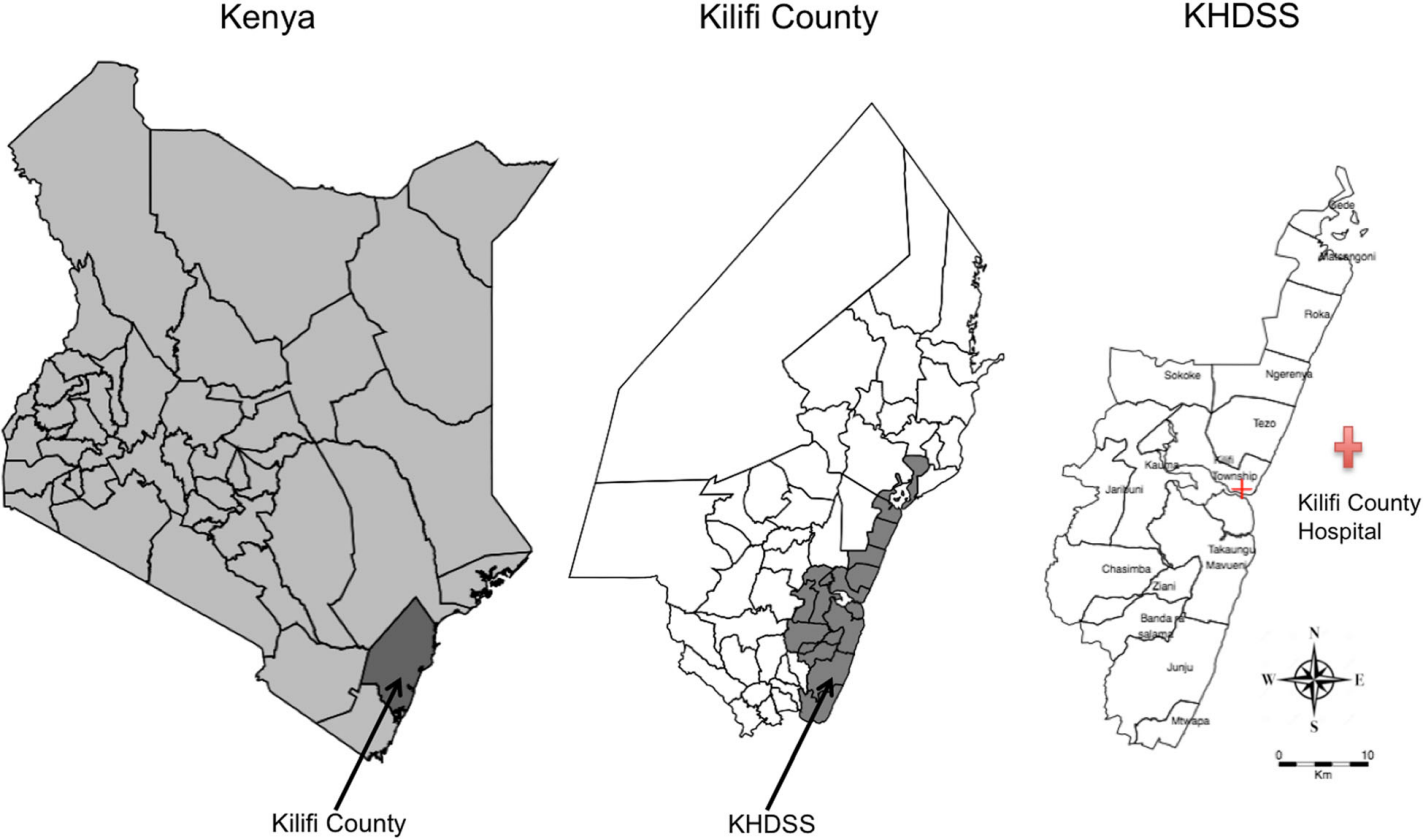
Map of study area. *Left*: location of Kilifi County in Kenya; *middle*: map of Kilifi County showing the boundaries of the KHDSS; *right*: map of the KHDSS showing the location of Kilifi County Hospital

Kilifi County stretches along 65 km of Kenyan coastline, extends 90 km inland from the coast at the widest point, and covers diverse ecological zones, Fig. 1. Malaria transmission increases after the long rains from April to June and the short rains from October to November each year, although transmission has been declining [15]. The county is subdivided into 36 administrative units known as locations. The Kilifi Health and Demographic Surveillance System (KHDSS), a member of the INDEPTH Network of demographic surveillance sites [18], covers an area of 891 km^2^ comprised of 15 administrative locations, containing 260,000 residents. The KHDSS area has been mapped using Magellan (Magellan Navigation Inc., Santa Clara, CA) and e-Trex (Garmin Ltd., Olathe, KS) Geographic Positioning Systems technology, providing detailed information on topography, footpaths and roads, and human occupation of the area, including the coordinates of all homesteads. Fieldworkers visit every participating household approximately three times a year to monitor births, deaths, and migration events. Kilifi County Hospital (KCH) is located centrally within the county; it provides primary care and serves as the first-level referral centre for the KHDSS area. For each paediatric inpatient admission to KCH, clinical examination, demographic details, laboratory analysis and outcomes are recorded in a central database. Every child admitted to the hospital is investigated with a thick or thin blood smear for microscopy to ascertain *Plasmodium* infection, regardless of presentation.

### Data collection

Data on bednet ownership and usage were collected during eight cross-sectional surveys integrated into the regular KHDSS enumeration in October 2008 – February 2009, March 2009 – August 2009, June 2010 – September 2010, May 2011 – September 2011, May 2012 – August 2012, May 2013 – October 2013, June 2014 – October 2014, and June 2015 – November 2015 (Additional file 1). The population of the KHDSS was the sample. Questionnaires were used to collect individual data on bednet ownership (i.e., was a bednet available for use in an individual’s usual sleeping area) and bednet use the night prior to enumeration. The response codes were “Yes”, “No” and “Don’t Know”. One resident was allowed to respond for all other residents of the same homestead.

Additional data on bednet ownership and use, as well as bednet observations, were collected as part of a separate cross-sectional survey, hereafter referred to as the sero-survey, that overlapped with the KHDSS enumeration survey (Additional file 1). The sero-surveys were conducted in June 2009 – September 2009, June 2011 – October 2011, June 2013 – October 2013, and June 2015 – October 2015 among randomly selected children aged 0–15 years who were residents of the KHDSS. For each round of the sero-survey, 500 children were selected at random from the KHDSS population register. Questionnaires (administered to the child’s parent/guardian) and field worker observations were used to collect individual data on bednet ownership, usage, whether bednet needed treatment with insecticide, number of nets, bednet characteristics (colour, brand and shape) and condition (number of holes and net hanging over the sleeping area).

From the pediatric admissions database, we extracted patient records of KHDSS children < 5 years admitted at KCH with a positive malaria slide any time during one of the eight KHDSS enumeration rounds. We excluded patients who were not KHDSS residents at the time of admission. We defined clinical malaria as a slide positive for *Plasmodium falciparum (P. falciparum)* parasites > 2500 parasites per μl [19]. We defined death related to malaria hospitalization as death that occurred during hospitalization among children with parasitemia and malaria as the discharge diagnosis.

### Statistical analysis

The proportions of individuals who owned and slept under a bednet the night prior to the survey were computed. The Z-test was used to test for a statistically significant difference of any two proportions over the eight-survey period. Spearman’s rank correlation was used to assess the association between bednet ownership and usage over the eight-survey period. The validity of the data collected during the KHDSS routine enumerations was assessed by comparison with observations collected during the overlapping sero-surveys. We performed a paired analysis on resident response compared to fieldworker observation and used McNemar’s test to test the consistency of the responses. The null hypothesis of the McNemar’s test was that there was no difference between reported bed net ownership and the observations of bednet ownership. We used Gwet’s AC1 statistic to assess the reliability of resident response vs. fieldworker observation of a bednet. Gwet’s AC1 (a chance-corrected agreement statistic) was used to quantify the extent of agreement between two paired observation as the surrogate for accuracy [20, 21]. Benchmarks for the strength of agreement have been constructed for its use to aid in communicability (< 0.20 poor agreement; 0.21–0.40 fair agreement; 0.41–0.60 moderate agreement; 0.61–0.80, good agreement; 0.81–0.99 very good agreement). Agreement was a measure of concordance between reported bednet ownership/usage and observing bednet i.e., for each paired observation, the proportion of reported and observed bednets classified into the same categories.

We used survival analysis to assess the effectiveness of bednet use in reducing malaria hospitalization over the eight KHDSS survey periods. The child years of observation under each period were computed using survival analysis methods. Age in years was used as the underlying time scale, with entry time defined as the start of each survey period or date of birth or date of in-migration, and exit time as 60 months of age, age at malaria diagnosis or death, or at end of the survey period or outmigration, whichever occurred first. The malaria hospitalization incidence rate was calculated as the number of admissions per 1000 child-years of observation. Two KHDSS surveys overlapped mass bednet distribution campaigns in the study community. To minimize misclassification of the exposure status (i.e., bednet ownership and use) arising from the July 2012 and October 2015 mass bednet distributions, we restricted the analysis to the survey period prior to the distribution campaigns. Given that the hospital malaria episodes were clustered within patients, we allowed for clustering by using a survival analysis with robust standard errors. The robust standard errors were used to account for the clustering effect in the estimation of the standard errors [22]. The ratio of malaria hospitalization in the non-bednet users to that in the bednet users was expressed as the hazard ratio (HR). With this ratio the overall percentage protective effect ((1 – HR) × 100) was obtained, which expressed the percent reduction in the overall hospitalization rate as a result of the bednet use. To estimate the number of hospitalizations averted [23, 24] we applied the hazard ratios obtained to the unexposed group. We adjusted the computed HR for sex, age, survey period and location of residence. Locations within the KHDSS were categorized as proximate (Kilifi Township) or distal (all the other locations except Kilifi Township) depending on the proximity to KCH, Fig. 1. The eight KHDSS surveys did not collect data on bednet quality, therefore, this was not considered in the analysis. Missing values on any of the variables of interest were excluded from the analysis. Multiple imputation has previously been used on this dataset with no significant difference between complete case analysis and multiple imputation [7]. A two-sided *P*-value of 0.05 was considered significant.

Data was stored in MySQL, cleaned and analyzed using STATA 13.1 (Stata Corp., College Station, TX, USA). The map was drawn using STATA and the Shapefiles downloaded from an open source website (https://africaopendata.org/dataset/kenya-counties-shapefile).

## Results

### Trends in bednet ownership and usage

**Fig. 2:**
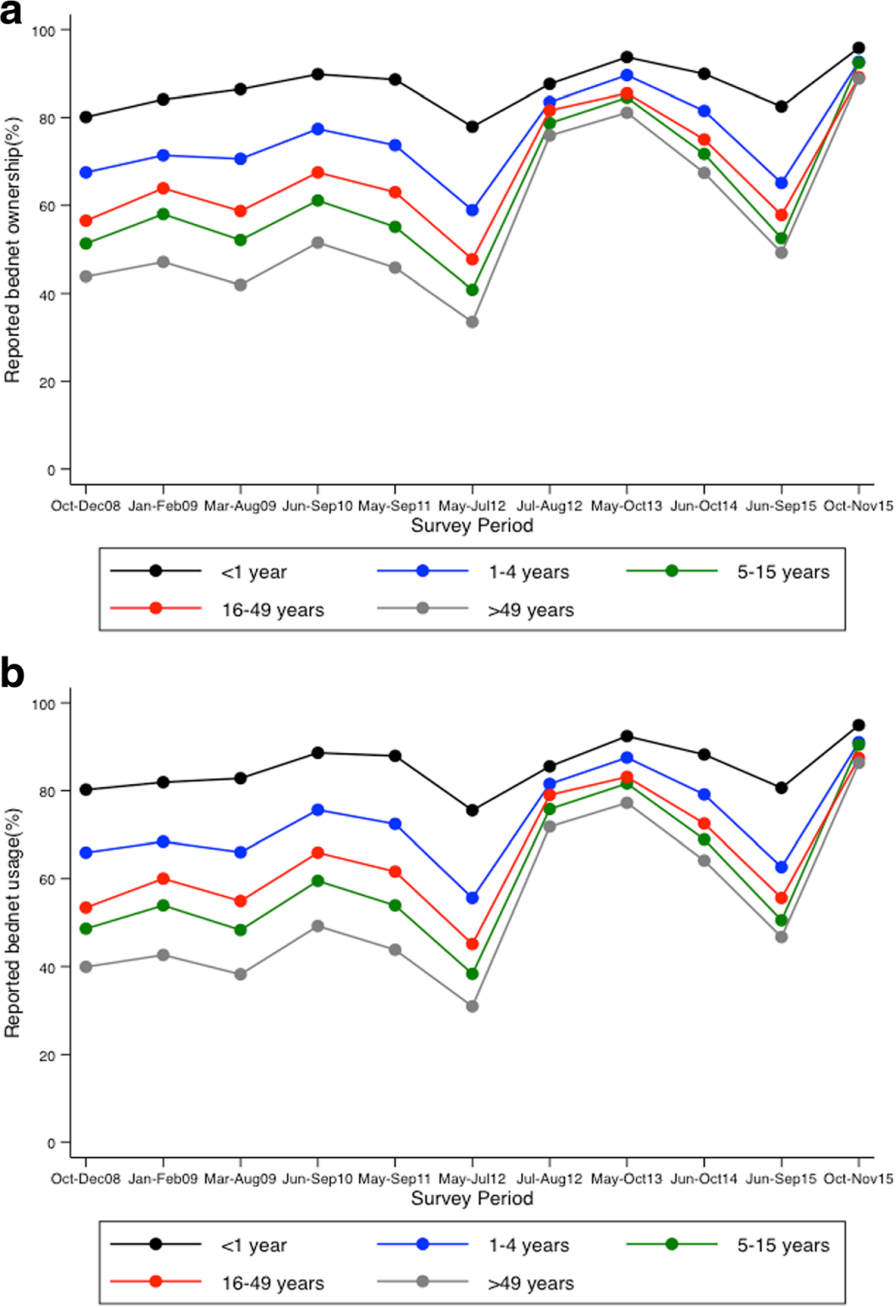
Change in bednet ownership and usage from 2008 to 2015. **a** Bednet ownership, and **b** Bednet use night prior to survey

**Fig. 3:**
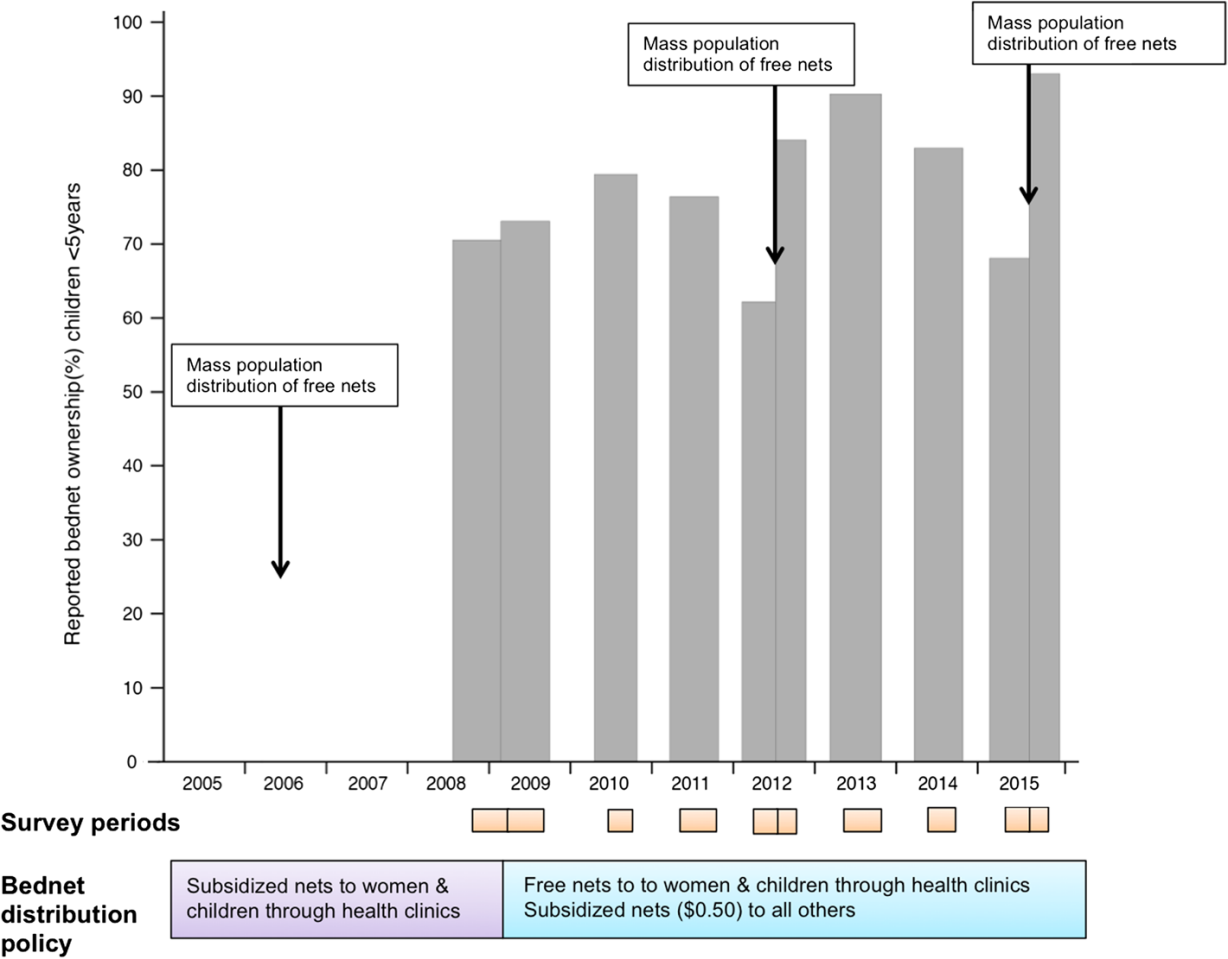
Coverage of bednet ownership in children < 5 years of age in 8 surveys over time and temporal relationship to bednet distribution policy

We observed 63% and 62% mean bednet ownership and usage, respectively, over the eight-survey period. There was a strong association between reported bednet ownership and usage between 2008 and 2015 (Spearman’s rho = 0.9498; *p* < 0.001). The overall proportion of bednet ownership in 2008 was 58% and in 2015 was 57% prior to the distribution campaign and 91% thereafter, with similar results observed for reported bednet usage (Fig. 2). Bednet ownership and usage were highest in infants (Fig. 2 & Additional file 2). Among children < 5 years, reported bednet ownership in October–December 2008 (i.e. prior to the change in policy to make bednets available free of charge) was 69% and in March–August 2009 was 73% (*p* < 0.001). An increase was also observed following the mass distribution campaigns in 2012 (62% in May–July 2012 vs 90% in May–October 2013; *p* < 0.001) and 2015 (68% in June–September 2015 vs 93% in October–November 2015; *p* < 0.001) (Fig. 3).

### Data validation: reported v/s observed bednets

Parents/guardians of 1986 children 0–15 years of age consented for their child to participate in the sero-survey study. Data on bednet ownership and bednet observation were available for 99% and 84% of these participants, respectively. A bednet was observed for 88% of sero-survey participants who reported owning a bednet in the KHDSS enumeration surveys. Among children < 5 years, the agreement between reported bednet ownership/use elicited during the KHDSS enumeration survey and the observation of a bednet during the sero-survey was > 75% (Gwet’s AC1 ≥ 0.59) for each of the four periods during which the KHDSS survey and the serosurvey overlapped (Table 1 & Additional file 3). The agreement between KHDSS reported ownership and observation of a bednet in each overlapping period was somewhat lower for older age groups, but was still > 65% (Table 1 & Additional file 3).

**Table 1:** Data validation: reported and observed bednet usage among KHDSS residents by survey period and age categories

		Bednet observed in sero-survey among reported bednet users in KHDSS			
Survey Period	Age group [years]	n	Proportion	Agreement [95% CI]	Gwet’s AC1
June – August 2009	0–4	283	88.5%	75.6% [70.2, 80.5]	0.59
	5–15	312	78.0%	68.9% [63.5, 74.0]	0.40
	Total	595	83.5%	72.1% [68.3, 75.7]	0.49
June – September 2011	0–4	172	82.9%	77.3% [70.3, 83.4]	0.60
	5–15	199	79.8%	73.4% [66.7, 79.4]	0.48
	Total	371	81.4%	75.2% [70.5, 79.5]	0.53
June – September 2013	0–4	161	98.6%	89.4% [83.6,93.7]	0.87
	5–15	188	91.7%	83.0% [76.8, 88.1]	0.77
	Total	349	94.9%	86.0% [81.9,89.4]	0.82
June – October 2015	0–4	149	92.8%	77.2% [69.6, 83.7]	0.67
	5–15	155	79.3%	67.1% [59.1, 74.4]	0.37
	Total	304	86.9%	72.0% [66.6,77.0]	0.52
Overall	0–4	765	90.7%	79.2% [76.2, 82.0]	0.68
	5–15	854	82.6%	72.7% [69.6, 75.7]	0.50

*n* number of individuals observed both in the KHDSS enumeration survey and sero-survey i.e. paired observations

Proportion = number of individuals for whom bednet was observed in the sero-survey divided by the number of individuals who reported bednet use during the KHDSS enumeration survey

*CI* confidence interval

### Reported bednet use and malaria hospitalization

Among children aged < 5 years, the overall malaria hospitalization rate was computed per 1000 child years of observation during each of the eight enumeration surveys (Table 2). The incidence of malaria hospitalization per 1000 child-years was 2.91 among children aged < 5 years who reported using a bednet the night prior to the survey and 4.37 among those who did not (HR = 0.67, 95% CI: 0.52, 0.85), Table 3. The incidence of death related to malaria hospitalization per 1000 child-years was 0.86 among children < 5 years who reported using a bednet the night prior to the survey and 1.73 among those who did not (HR = 0.45, 95% CI: 0.12, 1.66). In order to avert one case of malaria hospitalization 685 (95% CI: 507, 945) children needed to have used a bednet. When alternative definitions of malaria were used the estimates of the protective effect varied somewhat but were not significantly different from reported in Table 3 (Additional file 4). When the period of observation (i.e., the KHDSS survey period) was lengthened incrementally the protective effect was consistently lower but this was not statistically significant (Additional file 5).

**Table 2:** Rate of malaria hospitalization among children < 5 years in the KHDSS for each survey period

Survey period	Person- years	Malaria admissions, n	Rate per 1000 child-years	95% CI	
October 2008–February 2009	16,050	30	1.87	1.31	2.67
March–August 2009	15,945	43	2.70	2.00	3.64
June–September 2010	11,017	60	5.45	4.23	7.01
May–September 2011	13,015	47	3.61	2.71	4.81
May–July 2012	9495	20	2.11	1.36	3.27
May–October 2013	14,702	51	3.47	2.64	4.56
June–October 2014	12,292	60	4.88	3.79	6.29
June–September 2015	8056	44	5.46	4.06	7.34

*n* number of children admitted with malaria, *CI* confidence interval

**Table 3:** Incidence of malaria hospitalization & deaths related to malaria hospitalization among children < 5 years in the KHDSS by patterns of reported bednet ownership and usage

Outcome	Factor	Person-years	Malaria admissions, n^a^	Rate per 1000	Unadjusted HR^a^ (95% CI^a^)	HR^b^ (95% CI^a^)
Parasitemia >2500	Bednet owners	75,133	224	2.98	0.67 (0.53, 0.85)	0.67 (0.52, 0.87)
	Bednet non-owners	21,558	96	4.45		
	Bednet use last night	70,024	204	2.91	0.67 (0.52, 0.85)	0.67 (0.52, 0.86)
	Bednet non-use	23,098	101	4.37		
Death related to malaria hospitalization	Bednet owners	75,083	6	0.80	0.43 (0.12, 1.52)	0.38 (0.11, 1.27)
	Bednet non-owners	21,536	4	1.86		
	Bednet use last night	69,979	6	0.86	0.49 (0.14, 1.75)	0.45 (0.13, 1.55)
	Bednet non-use	23,075	4	1.73		

^a^ *HR* Hazard Ratio, *CI* confidence interval, ^b^The HR was adjusted for sex, age, location of residence and period of the survey

## Discussion

Malaria remains a major cause of morbidity and mortality, particularly in sub-Saharan Africa. Many countries, including Kenya, have attempted to reduce the burden of malaria through policies and practices that promote bednet ownership and use. We report that bednet ownership and use remained relatively stable over the 8-year period and temporarily increased corresponding to mass distribution campaigns. We found that bednet use was associated with a 33% reduction in the incidence of malaria hospitalization among children < 5 years.

Pooled data from controlled trials of insecticide treated nets have consistently demonstrated reductions in mortality, morbidity and malaria parasitemia of 17, 50 and 13%, respectively [6, 25]. Although a number of studies have postulated a causal relationship between bednet use and reductions in childhood morbidity and mortality, there is little data on the protection against malaria hospitalization afforded by bednet use under routine conditions. Our estimate of the protection against malaria hospitalization associated with bednet use is in agreement with a randomized controlled study conducted in the study area that documented a 42% (95% CI; 21, 57) protective efficacy against severe malaria [14]. It has been estimated that substantial protective community effects of net ownership are observed with coverage ≥ ~ 50% [7, 26, 27]; in the study area overall bednet ownership/usage was observed to be > 50% over the 8-year period. Bednet use was also associated with a reduction in deaths related to malaria hospitalization although this was not statistically significant. The number of malaria-related deaths was low; this may reflect local care-seeking and case-management practices rather than bednet use ownership and use.

The protective association of bednet use has been shown to vary by setting, making a multi-faceted approach to malaria control essential [28]. In our setting, bednet usage was highest among children < 5 years and women of reproductive age [29]. This pattern of bednet use has been observed in other areas in Africa [30–32] and likely reflects population sub-groups targeted by net distribution campaigns. Bednet usage among women of reproductive age was 60% in the 2008/2009 KHDSS survey – similar to the 58% usage reported in a 2007 survey in western Kenya [17] - and increased to 89% in 2015 following a distribution campaign.

Observing a mounted bednet is considered a more reliable indicator of bednet use than relying solely on self-reported use; however, the time required to do a thorough assessment of the household, along with local customs that typically prohibit strangers from entering the household, can preclude conducting such surveys at scale. In a contemporaneous survey of a randomly selected subset of children under 16 years in the KHDSS, we observed a bednet in 86% of children for whom net ownership was reported as “yes”. This suggests that self-reported net-use the night prior to the survey may overestimate actual net use to a small degree in our setting but is a reasonable proxy. The median time lapse between the routine KHDSS enumeration rounds and sero-study interviews was 1 month (IQR: 0.4, 2.5). We considered the time lapse to be reasonable to make a comparison between observed and reported bednet ownership and usage; however, the gap in time may have contributed to the lack of perfect agreement in the data.

Our study has several other limitations. Because we did not require each resident to be present to respond to the questions, data on bednet ownership and usage may have been incorrectly reported. We attempted to minimize this potential misclassification by instructing fieldworkers to interview only residents of the same homestead regarding bednet ownership and usage. Although non-significant, there was a trend toward a reduced protective effect as the period of observation was lengthened 1, 2, and 3 months before and after the KHDSS enumeration period. This suggests that limiting the analysis to the KHDSS enumeration periods was an appropriate choice to minimize misclassification of exposure status that could arise due to variation in bednet use or ownership over time. In this study, we asked about individual bednet ownership – defined as availability of a bednet for an individual’s usual sleeping space – and bednet use the night prior to enumeration. This limits comparability to studies that report ownership at a household level. While we found good agreement between bednet ownership reported during the KHDSS routine enumerations and bednet observation during the sero-survey, this does not directly validate reported bednet usage estimates. The results on the incidence of malaria hospitalization may be biased and confounded by other unmeasured factors (e.g., variation in housing, urbanization, socio-economic status, mother’s education, care-seeking behaviors). Therefore, the estimates obtained could be an overestimation or underestimation of the true incidence. Because we included all bednets, regardless of quality or insecticidal efficacy, we likely underestimated the protection afforded by usage of a high-quality, long-lasting insecticide treated net.

The strengths of the study include use of demographic surveillance data of a sizeable population, the longitudinal nature of the study, which allowed us to evaluate trends in bednet ownership and use over time in association with changes in government policy on provision of bednets and mass distribution campaigns, and the validation of the demographic surveillance data using the sero-survey data. In addition, the diagnostic and laboratory evaluation of children admitted to KCH was the same throughout the study period.

## Conclusion

Bednet use was associated with a 33% reduction in the incidence of malaria hospitalization in children < 5 years. On longitudinal surveillance, we observed increasing bednet ownership and usage following mass distribution campaigns. A high proportion of people who reported owning a bednet also reported using a bednet, suggesting that efforts to increase net ownership would result in increased net usage. Ownership and usage declined in the years following mass distribution campaigns; to achieve and sustain universal coverage, expansion of continuous distribution channels or other distribution methods should be considered.

## Additional files

Additional file 1: Timeline for the KHDSS bednet survey, sero-survey and the mass distribution. (DOCX 260 kb)

Additional file 2: Baseline characteristics, bednet ownership and usage, and hospital admission among residents of KHDSS during 8 rounds of enumeration. (XLS 76 kb)

Additional file 3: Data validation: reported and observed bednet ownership status among KHDSS residents by survey period and age categories. (XLS 37 kb)

Additional file 4: Sensitivity analysis: Incidence of malaria hospitalization among children < 5 years in the KHDSS by pattern of reported bednet ownership and usage, defining malaria three different ways. (XLS 37 kb)

Additional file 5: Sensitivity analysis: Incidence of malaria hospitalization (parasitemia > 2500) among children < 5 years in the KHDSS by pattern of reported bednet ownership and usage, using incrementally longer periods of observation. (XLS 39 kb)

## Abbreviations

HR: Hazard ratio
KCH: Kilifi County Hospital
KHDSS: Kilifi Health and Demographic Surveillance Site
OR: Odds ratio
*P. falciparum*: *Plasmodium falciparum*
