# Supplementary figures and images for: Trends in bednet ownership and usage, and the effect of bednets on malaria hospitalization in the Kilifi Health and Demographic Surveillance System (KHDSS): 2008–2015
Source: BMC Infect Dis. 2017 Nov 15;17:720. doi: 10.1186/s12879-017-2822-x (PMC5688631; doi:10.1186/s12879-017-2822-x)

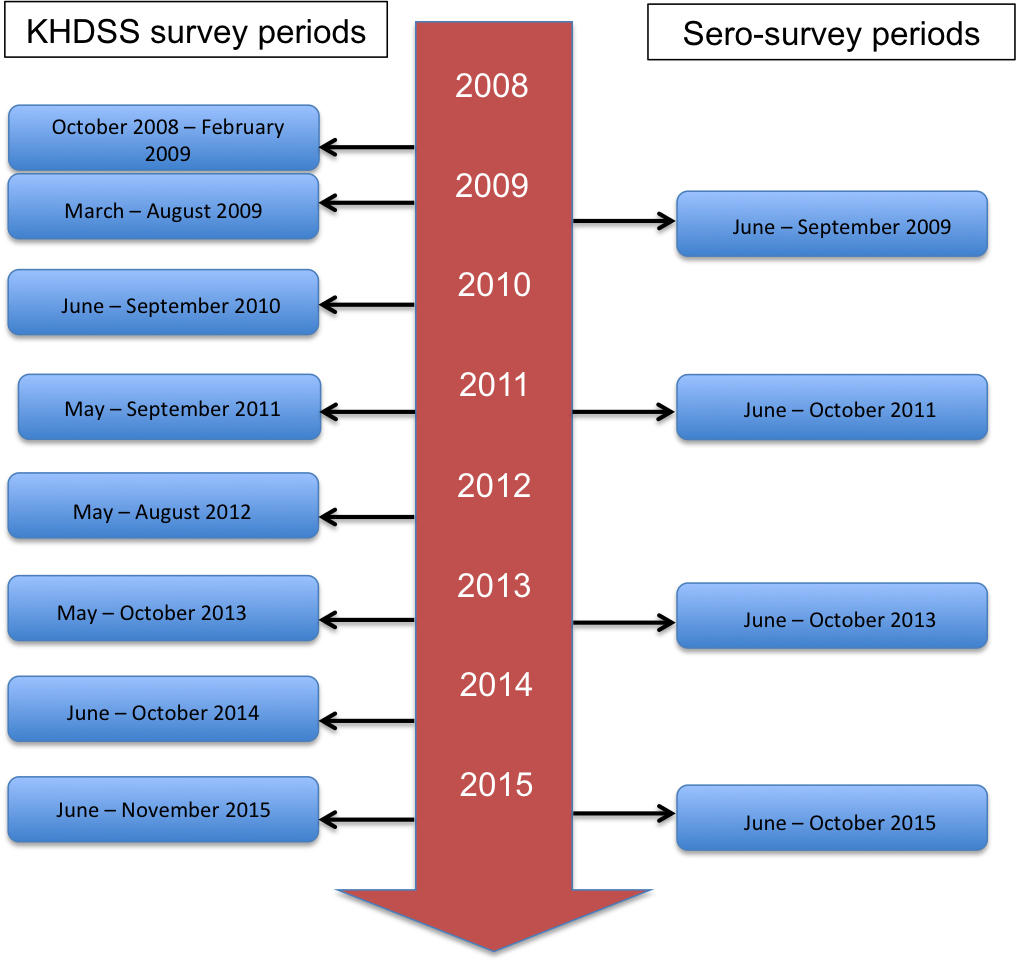

Supplement: Supplementary file 1 — Timeline for the KHDSS bednet survey, sero-survey and the mass distribution. (DOCX 260 kb) [file 12879_2017_2822_MOESM1_ESM.docx]
